# An assessment of the amount of untapped fold level novelty in under-sampled areas of the tree of life

**DOI:** 10.1038/srep14717

**Published:** 2015-10-05

**Authors:** Daniel Barry Roche, Thomas Brüls

**Affiliations:** 1 Laboratoire de Génomique et Biochimie du Métabolisme, Genoscope, Institut de Génomique, Commissariat à l’Energie Atomique et aux Energies Alternatives, Evry, Essonne, 91057, France; 2 UMR 8030 – Génomique Métabolique, Centre National de la Recherche Scientifique, Evry, Essonne, 91057, France; 3 Départment de Biologie, Université d’Evry-Val-d’Essonne, Evry, Essonne, 91000, France; 4 PRES UniverSud Paris, Saint-Aubin, Essonne, 91190, France; 5 Institut de Biologie Computationnelle, LIRMM, CNRS, Université de Montpellier, Montpellier, 34095, France; 6 Centre de Recherche de Biochimie Macromoléculaire, CNRS-UMR 5237, Montpellier, 34293, France

## Abstract

Previous studies of protein fold space suggest that fold coverage is plateauing. However, sequence sampling has been -and remains to a large extent- heavily biased, focusing on culturable phyla. Sustained technological developments have fuelled the advent of metagenomics and single-cell sequencing, which might correct the current sequencing bias. The extent to which these efforts affect structural diversity remains unclear, although preliminary results suggest that uncultured organisms could constitute a source of new folds. We investigate to what extent genomes from uncultured and under-sampled phyla accessed through single cell sequencing, metagenomics and high-throughput culturing efforts have the potential to increase protein fold space, and conclude that i) genomes from under-sampled phyla appear enriched in sequences not covered by current protein family and fold profile libraries, ii) this enrichment is linked to an excess of short (and possibly partly spurious) sequences in some of the datasets, iii) the discovery rate of novel folds among sequences uncovered by current fold and family profile libraries may be as high as 36%, but would ultimately translate into a marginal increase in global discovery of novel folds. Thus, genomes from under-sampled phyla should have a rather limited impact on increasing coarse grained tertiary structure level novelty.

The structure-function relationship forms the basis of a molecular description and understanding of the properties and cellular roles of proteins. Nowadays, protein 3D structure determination remains essentially a low to medium throughput process, while sequencing has entered a high-throughput regime in the last decade. As a result, sequence based structure inference methods rely on existing structural knowledge and remote homology detection techniques are the *de facto* means to infer structural properties of a protein sequence^1^.

Therefore, global structural genomics (SG) efforts aim at producing a representative set of folds to act as templates in homology modelling analyses, thereby providing functional insights for currently unannotated sequences^2^,^3^,^4^,^5^,^6^,^7^,^8^,^9^,^10^. Because the frequency distribution of different folds is highly skewed, this goal branched into two almost antithetic sub-goals of i) maximizing function prediction through structure based detection of homology, and ii) closing in on a complete set of folds.

The first of these two goals aims to expand the structural coverage of protein families, monitoring the fraction of sequence space that has been covered (i.e. structurally characterized) as structural genomics efforts proceeded^11^.

This aim of increasing the structural coverage of protein sequence space has led to protocols for targeting representatives from large, structurally uncharacterized domain families, as well as members from very large and diverse families with partial structural coverage, as such large and diverse families (both structurally and functionally) are highly overrepresented in known genomes^3^,^4^,^5^.

Several metrics, e.g. “modelling leverage” that measure the number of reliable comparative models that can be built from each experimental structure (see also the “novel leverage”^7^,^9^ metric) have monitored the benefits resulting from a systematic targeting of large families.

It is however increasingly difficult to achieve novel leverage over time; as noted by Liu *et al*.^7^ and Nair *et al*.^9^, although the number of structures has increased exponentially, the structural coverage of sequence databases has increased only linearly (it should be stressed however that structural genomics efforts have been instrumental in maintaining this growth rate^9^).

In that respect, it is noteworthy that nowadays protein families (Pfam^12^) are characterized at the lowest rate in almost two decades. Already in 2006, Chandonia and Brenner^6^ noted that the rate of first structural characterization of families rose steadily throughout the 1990 s but had levelled off at around 20 new families per month since 1999, even as the total number of structures solved continued to increase.

In a more recent study, Mistry *et al.*^13^ measured that, for every 100 new PDB entries an estimated 20 Pfam families acquired their first structure twenty years ago, while by 2012, the figure decreased to only about five families per 100 structures^13^.

Among the reasons lies the fact that the set of structurally uncharacterized Pfam families are smaller in size, functionally uncharacterized (i.e. enriched in domains of unknown function (DUFs)), and enriched in problematic features like transmembrane and intrinsically disordered regions^13^.

Another source of structural bias in current databases is related to the high proportion of new structures that are solved by molecular replacement after the first structure in a family is solved.

Nevertheless, the focus on structural coverage of sequence space has enabled the routine assignment of about two thirds of the sequences from completed genomes to structurally represented domain families, thus enabling ancient evolutionary relationships to be inferred^14^.

Here, we focus on the second goal evoked above, i.e. achieving full coverage of protein fold space. As previous studies suggested that structural coverage of folds may be reaching a plateau^5^,^6^, we wanted to assess whether phylogenetic biases in the choice of targets for genome sequencing could have propagated at the structure level and resulted in a corresponding undersampling of protein fold space, and whether untapped sources of structural novelty can be expected in currently undersampled regions of the tree of life.

Background supporting this hypothesis is provided by previous studies^2^,^5^,^6^,^8^ documenting that structural genomics centres with highest novelty rates in sequence-based tests also had the highest rates of novelty in structure based hierarchies.

In 2000, Brenner and Levitt^2^ noticed that fewer than a quarter of proteins (domains) lacking significant pairwise sequence similarity to those already in the protein database had a new fold, compared with about a half in 1990. Hence, they expected that about a quarter of the early structural genomics targets would have a new fold, and suggested that by avoiding the targeting of homologs of known structures, structural genomics centres might increase the percentage of new SCOP^15^ folds and superfamilies discovered to about 40%^2^.

However, in 2006, Chandonia and Brenner^6^ analysed the production of Protein Structure Initiative (PSI) centres and measured that the percentage of domains representing a new SCOP fold or superfamily was only 16%, a figure higher than the 4% figure from non-structural genomics centres, but nevertheless much lower than the expectation of 40%^6^.

In an independent analysis of 323 protein chains corresponding to 459 CATH and 393 SCOP domains, Todd *et al*.^5^ observed that 10% and 11% of these contributed new folds in SCOP and CATH respectively. These observed depletions of high level structural novelty raised the possibility that part of the observed fold saturation is provoked by biased sampling of the tree of life, as most targets for structure elucidation were picked up from genomic sequences.

On one hand, the taxonomic composition of sequence databases has dramatically changed over the last five years, and these changes are effectively redefining the scope and contribution of large-scale structural efforts^11^. For example, the faster-growing bacterial genomic entries have overtaken eukaryotic entries, leading to an increase in redundancy^11^.

On the other hand, metagenomic initiatives have dramatically increased our knowledge of the protein universe, e.g. by providing the majority of currently known protein sequences, including thousands of new families of unknown function, while at the same time also increasing phylogenetic diversity within the sequence databases^16^,^17^.

However, structure determination and analysis of 250 representatives of such novel families, led Godzik and colleagues^18^ to conclude that most of the families represented distant homologs of already characterized protein families, suggesting that the bulk of protein diversity within the metagenomic dataset they studied resulted from functional divergence among already known protein families^18^.

A similar impression emerged from a structural survey of DUFs (including a large set of metagenomic origin) by Jaroszewski *et al*.^19^, as the authors concluded that about two thirds of the DUF families likely represent very divergent branches of already known and well-characterized families. Hence, these studies inferred that, despite the tremendous inflation in protein sequence space enabled by high throughput sequencing technologies, the corresponding fold space of the proteins is reaching saturation.

Nevertheless, and importantly, by showing that about one third of the DUF families could be formally categorized as new folds^19^ -compared to the 10% to 16% fold novelty rate measured in Todd *et al*.^5^ and Chandonia and Brenner^6^, together with a similar figure for the metagenome derived families studied by Godzik^18^, these analyses confirmed that DUF families and metagenomes could constitute a promising reservoir for the discovery of new folds and topologies.

Sustained technological developments enabling *en masse* sequencing of microbial communities as well as single cell sequencing of uncultivated micro-organisms thus seem to have the potential to be a source of novelty at both sequence and 3D structural levels.

Building upon these earlier studies, we pursue this investigation in a complementary way to assess whether access to under-sampled areas of the tree of life that is enabled by these technologies, and specifically the genomic sequences generated within the Microbial Dark Matter (MDM) initiative^20^, within a community genome analysis of an acetate-amended aquifer^21^, as well as genomes targeted within the Human Microbiome Project (HMP)^22^, could add to overall fold space diversity.

## Methods

### Variations in proteome coverage by fold and domain family libraries

For our analysis, we selected recently characterized genomes (mostly from uncultured organisms) from under-sampled regions of the tree of life, as we hypothesized that these organisms have the potential to code for novel proteins at both a sequence and structural level.

We performed a sequence based structural survey of 41 phylogenetically diverse (encompassing 12 distinct phyla and comprising 147,315 proteins) bacterial genomes from the GEBA initiative^23^ and of more than six hundreds genomes that were reconstructed from uncultured candidate phyla using either metagenomics (47 genomes - 48,839 proteins - recovered from an acetate amended aquifer (AAA)^21^) or single cell based techniques (555 genomes (460,519 proteins) recovered from the “Microbial Dark Matter” (MDM) project^20^). These were complemented with 195 genomes (comprising 943,545 proteins) from the Human Microbiome project (HMP)^22^ as well as an *E. coli* pan-genome featuring 62 distinct genomes containing 289,457 proteins (note that different *E. coli* strains can differ by up to 1/3 of their genome length). In total, we surveyed 1,838,675 proteins.

Standard fold recognition techniques were used to predict coarse grained protein structure and CATH^24^ family membership. HMMER’s hmmscan algorithm^25^ was used along with the CATH/Gene3D fold library^26^ and an e-value cutoff of 10^−5^. In order to enable the determination of Pfam family^12^ membership, HMMER’s hmmscan^25^ was used along with the Pfam27A library, and standard cutoffs calibrated for each individual PfamA family were used to filter positive hits.

Intrinsically disordered proteins were predicted using the DisEMBL^27^ algorithm with standard parameters. A protein was determined to be intrinsically disordered if 50% or more of its residues were predicted to fall into disordered regions.

Trans-membrane proteins were identified using the TMHMM^28^ algorithm with standard parameters. Proteins were determined to be transmembrane associated if the target sequence contained 3 or more transmembrane helices that made up at least 50% of the length of the target sequence.

The distribution of the sequences within each of the above categories were calculated, as well as the overlap between categories, e.g. between fold and protein family based assignments (Supplementary Table S1 to Table S5).

In order to increase the sensitivity of the analysis, HHblits^29^ -a profile-profile based alignment algorithm- was additionally used to screen the unassigned sequences against the PfamA and SCOP fold libraries. Sequences scoring a top hit with a p-value < 10^−5^ were deemed to be true positive hits.

In order to assess possible sequence length biases, the length of protein sequences with and without associated assignment was compared for each dataset (Supplementary Figures S2–S6). An excess of short sequences was apparent in some datasets, which provoked us to recalculate the different assignment categories (i.e. fold, Pfam, disordered and transmembrane segments) after discarding sequences less than 100 amino acids in length.

### Relating Pfam sequence novelty and fold level structural novelty

We then derived an empirical relationship between sequence level and structure level novelty, using the Pfam5000^4^ (i.e. the largest Pfam families present across releases 20 to 27) and Pfam_new (see below) datasets, to estimate how observed sequence novelty might translate into high level structural novelty.

Briefly, Pfam releases 20 to 27 were downloaded from the Pfam^12^ ftp site along with the descriptions of changes that occurred between each release. Using the latter, the new families for each release were identified (referred to as Pfam_new in the remainder of the text), while families deleted between releases were tracked and excluded from the analysis. Two subsets, Pfam5000LARGE and Pfam5000SMALL, were also defined as the 5000 largest and 5000 smallest Pfam families present in releases 20 through 27.

Next, we identified the first structure that was solved for each Pfam family by mapping the release date of each protein structure from PDBsum^30^ to each Pfam family; the structure with the oldest release date being identified as the first structure for that Pfam family. A file containing a mapping of Pfam family IDs to PDB IDs for Pfam release 27^12^ (recovered from the Pfam web site) enabled us to attach a date to the first structure released from each Pfam family, allowing us to determine if it was anterior or posterior to the Pfam release.

In order to contrast the rate of new fold discovery occurring in all Pfam families *versus* the rate measured in the subset of new Pfam families (Pfam_new), we compared the first structure of all families that acquired a structure between Pfam 20 and Pfam 27 to a non-redundant (using a 90% sequence identity criterion) PDB library only containing structures anterior to Pfam release 20, thus embedding the structural knowledge available at that time. The rationale behind this operation is that we hypothesized that the subset of domains assembled in Pfam_new should behave similarly in terms of structural novelty as the currently uncharacterized (i.e. not covered by current domain family libraries) sequences identified in the genomes analysed here.

A protein was determined to have a new fold if the score computed with the TM-align algorithm^31^ between the target protein and all template proteins in the non-redundant PDB library was less that 0.5 (we actually checked that the results were robust with respect to varying the threshold across the 0.4 to 0.6 interval). Such TM-scores ranging from 0.4 to 0.6 have previously been shown (by extensive benchmarking using the SCOP and CATH structural classifications) to represent accurate thresholds to separate related and unrelated folds^32^, and are commonly used by numerous fold recognition methods, model quality assessment programs and structure-based ligand binding site predictors. This analysis was carried out for both the Pfam5000 (large and small) and Pfam_new datasets.

## Results

### Apparent increase of sequence novelty in proteomes from under-sampled phyla

**Figure 1:**
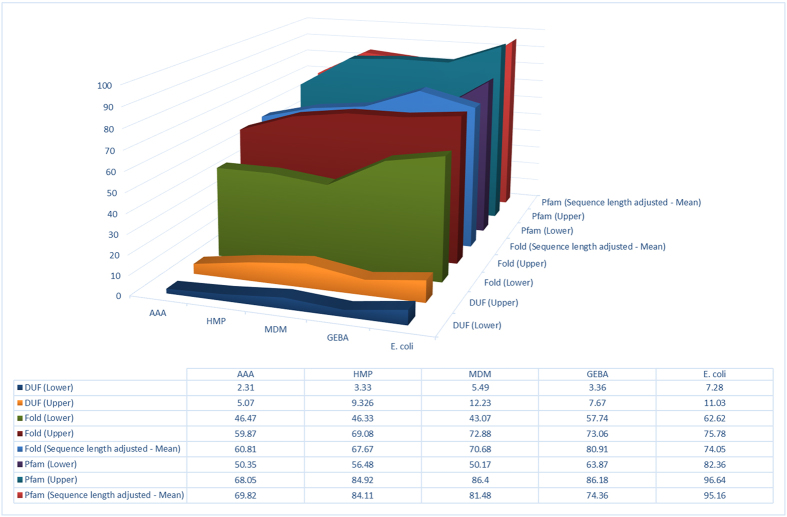
Variation in proteome coverage by fold, Pfam and DUF profile libraries between the different datasets (see text). Upper and lower limits correspond to most extreme genome level variation within a dataset. For the sequence length adjusted datasets the mean score is shown.

The fraction of the different proteomes that can be confidently assigned to at least one fold or Pfam family turned out to be significantly lower (Mann-Whitney test, p < 1e-5, Supplementary Table S6) in the MDM, AAA and HMP datasets, compared to the coverage achieved in both the set of phylogenetically diverse GEBA genomes and the *E. coli* pan-genome (Fig. 1). For example, the proportion of proteome devoid of hits to both the fold profile library and Pfam families was as high as 32% in three distinct phyla from HMP, 5 phyla from MDM and one phylum from AAA, and reached a minimum of 2% in one *E. coli* genome (Supplementary Table S1 to Table S5).

The observed higher proteome proportions without sequence family anchoring or structural type assignment in proteomes from under-sampled phyla cannot be accounted for by higher occurrence of problematic features like internally disordered segments or transmembrane regions (Supplementary Table S1 to Table S5), neither by a lack of sensitivity in the fold recognition methods that were used (see below). Overall, this lack of genomic coverage with respect to Pfam and fold libraries is compatible with untapped sources of either sequence novelty alone (i.e. without associated structure level novelty) or combined sequence and structure novelty. Borrowing terminology from epistemic modal logic, we could refer to the former as “known-unknowns” and the latter as “unknown-unknowns”, while the sequences covered by the fold and domain libraries would constitute the “known-knowns”.

### Estimation of the fold-level novelty rate in “uncovered” sequences

To disentangle these two distinct sources of novelty and estimate the amount of structural novelty to expect from sequences that are not yet covered by Pfam profiles, we measured the amount of new folds that arose from a set of first structurally solved members of newly created Pfam families over time (see Methods), and compared this value with the amount of fold level structural novelty that arose from Pfam5000 (which was used for target selection in structural genomics efforts).

We measured that about 36% of the Pfam_new families gave rise to new folds between Pfam20 and Pfam27 releases (Supplementary Figure S1), whereas the new fold discovery rate measured in all Pfam families between Pfam20 and Pfam27 was much lower (2.6%), similar to the rate for structures derived from Pfam5000 (1.5%). The latter figure is much lower than the 10% to 16% measured in Todd *et al.*^5^ and Chandonia and Brenner^6^, but consistent with statistics computed from current PDB/SCOP releases (http://www.rcsb.org/pdb/statistics/contentGrowthChart.do?content=fold-cath), and opportunely illustrates the drop in new fold discovery that occurred in the last five years. On the other hand, our 36% figure is consistent with the pioneering analyses of Godzik^18^ and Jaroszewski *et al.*^19^.

The difference in novel fold discovery rates between proteins whose sequences are already covered by known protein domain families (including domains of unknown function, DUFs) and those which are not, suggest that significant amounts of fold novelty could reside in the genomes from under-sampled phyla.

### Variation in proteome coverage is not explained by lack of sensitivity of fold recognition methods (i.e. sequence vs HMMs)

To assess whether coverage differences could be attributed to a lack of sensitivity of sequence-HMM *versus* HMM-HMM based methods, HHblits profile-profile based alignments were generated for all unassigned sequences. This only resulted in a negligible increase of fold assignment from 0.33% (1,520/460,519 proteins) for the Microbial Dark Matter (MDM) dataset to 1.38% (13,021/943,545 proteins) for the Human Microbiome Project dataset. The use of a profile-profile based method also increased Pfam family assignments, ranging from 0.60% (1,737/289,457 proteins) for the *E. coli* pangenome to 10.48% (98,884/943,545 proteins) for the Human Microbiome Project dataset. Regarding the latter figure, it should be noted that, as there were no family-specific calibrated cutoff scores available for the HHblits Pfam library, Pfam assignments were carried out using a single cutoff of 10e-5, which probably resulted in higher assignment levels.

### Variation in proteome fold coverage is linked to an excess of short length gene predictions in genomes reconstructed from uncultured organisms

The variation in fold level coverage can be linked to an excess of short sequences in some datasets (See Supplementary Figures 2 to 6) (New dataset sizes; AAA = 39,757 proteins; GEBA = 123,956 proteins; MDM = 50,161 proteins; HMP = 751,203 proteins; Ecoli = 253,973 proteins). Both the fold and Pfam domain family coverage increased when excluding sequences less than 100 amino acids in length from the analysis: fold assignments increased by up to ten percent, while Pfam based assignments increased by up to seven percent (Fig. 1), highlighting the ambiguous and possibly spurious nature of short and very short coding sequences predicted in some culture-independent datasets. Crucially, the combined fold and Pfam coverage no longer differed between the datasets after exclusion of all the sequences shorter than 100 residues, even though the overall level of assignment (which also includes assignments to the transmembrane and disordered categories) did not differ significantly between the original and length filtered datasets. It is also noticeable that the AAA dataset still remains an outlier after the filter, as it displays a markedly lower percentage of assigned sequences compared to the other datasets, the latter having over 85% (GEBA = 105,323/123,456 proteins; HMP = 646,260/751,203 proteins; MDM = 44,754/50,161 proteins; Ecoli = 243,586/253,973 proteins) of their sequences assigned to at least one of the categories. With this respect, it could be worth noting that the original assemblies for the AAA dataset were of draft quality and quite fragmentary (as assessed by marker gene content analysis, data not shown), which could affect proteome quality. Unfortunately, we could not recover the original contigs for further analyses.

### Conclusion: increased sequence novelty but limited fold level structural novelty in genomes from under-sampled phyla

**Figure 2:**
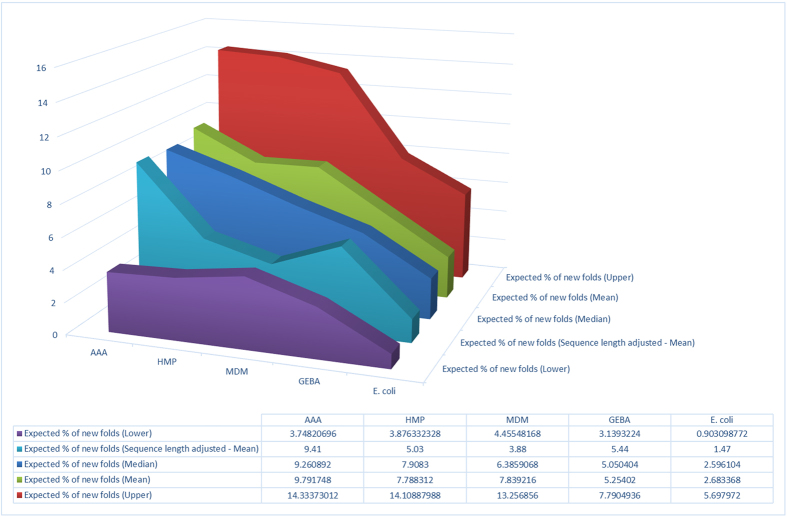
Expected rates of novel fold discovery for the different datasets (see text).

Assuming the discovery rate of novel folds among Pfam_new is a reasonable approximation for fold novelty among uncharacterized (i.e. neither covered by Pfam nor fold profile libraries) sequences, and combining this figure with the observed fraction of such sequences in genomes from under-sampled phyla *versus* those from more deeply sampled areas of the tree of life, leads us to estimate that the overall amount of fold level novelty extractable from the former genomes is only marginally higher, with the possible exception of the ambiguous AAA dataset (Fig. 2). Thus, the present analysis suggests that, even though under-sampled regions of the tree of life can significantly increase coverage of protein sequence space, this in turn, according to the empirical relationship between sequence and structure novelties measured here, leads to only a modest increase of protein fold space sampling.

Nevertheless, because efforts for sequence-based characterization of uncultured phyla are at an early stage, it is still plausible that the fraction of structures solved that are novel at high level could benefit from enhancing the sequence based target selection protocol with a more careful genome selection protocol, e.g. by actively targeting uncharacterized proteins from uncultured organisms accessed via single cell, metagenomics and yet to come sequence technology developments.

## Additional Information

**How to cite this article**: Barry Roche, D. and Brüls, T. An assessment of the amount of untapped fold level novelty in under-sampled areas of the tree of life. *Sci. Rep.* **5**, 14717; doi: 10.1038/srep14717 (2015).

## Supplementary Material

Supplementary Information
