# Supplementary material for: An assessment of the amount of untapped fold level novelty in under-sampled areas of the tree of life
Source: Sci Rep. 2015 Oct 5;5:14717. doi: 10.1038/srep14717 (PMC4592975; doi:10.1038/srep14717)

An assessment of the amount of untapped fold level structural novelty in under-sampled areas of the tree of life

Daniel Barry Roche[1,2,3,4,5,6*]
Thomas Brüls[1,2,3,*]

[1]Laboratoire de Génomique et Biochimie du Métabolisme, Genoscope, Institut de Génomique, Commissariat à l'Energie Atomique et aux Energies Alternatives, Evry, Essonne, 91057, France

[2]UMR 8030 – Génomique Métabolique, Centre National de la Recherche Scientifique, Evry, Essonne, 91057, France

[3]Départment de Biologie, Université d'Evry-Val-d'Essonne, Evry, Essonne, 91000, France

[4]PRES UniverSud Paris, Saint-Aubin, Essonne, 91190, France

[5]Institut de Biologie Computationnelle, LIRMM, CNRS, Université de Montpellier, Montpellier, 34095, France

[6]Centre de Recherches de Biochimie Macromoléculaire, CNRS- UMR 5237, Montpellier, 34293, France

SUPPLEMENTARY DATA

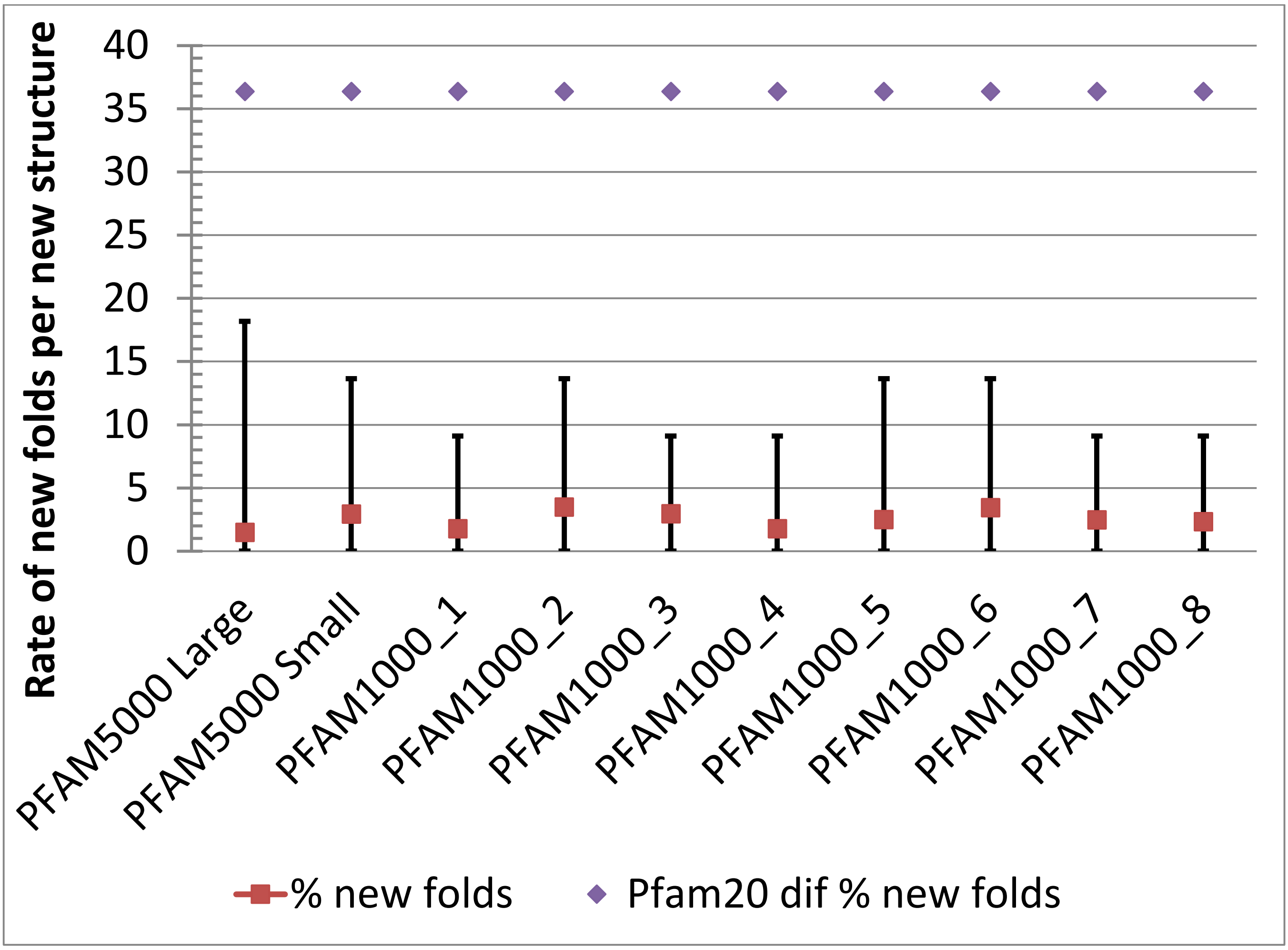

Figure S1: Rate of new fold occurrence in different Pfam datasets (see text).

Table S1: Assignment of sequences into each category of fold, Pfam, disorder as well as transmembrane is shown for the MDM dataset.

Phyla	*% Fold*	*% Pfam*	*% Disorder*	*% TM*	*% Overlap – Fold and Pfam*	*% Assigned*
Armatimonadetes_bacterium_JGI	71.41099	77.03035	10.0902379	10.33634	65.7916325	85.6029532
BRC1	61.96476	69.24513	6.48699102	10.71054	56.4694901	77.6572162
CD12	67.68542	77.06833	11.1641036	10.6969	63.5098306	84.0373759
DHVE3	60.82949	70.3533	16.0522273	9.3702	56.8356375	77.8033794
DUSEL1	47.20735	51.95103	17.7505738	8.11018	42.310635	62.5095639
DUSEL2	49.5372	55.29654	13.6784368	10.69592	44.1549537	64.4154954
DUSEL3	52.28731	58.8491	14.5725523	9.41834	47.4642931	69.2610226
DUSEL4	48.72152	52.87773	18.2705718	6.68526	43.4402603	64.4723384
EM	65.49112	73.49846	9.75773716	9.95165	60.906999	81.1541743
GN02	43.07158	50.1659	16.4796966	5.5459	38.8686996	60.5782904
KSB	63.49283	74.01788	7.21893206	8.98356	59.5908669	79.7556298
NKB19	69.37128	79.01758	7.48443593	10.30991	65.1843744	85.6605323
NPFFA	64.30886	74.00586	9.40377591	12.86589	61.2119965	79.3610782
OD1	62.2778	69.51085	14.6254751	10.71472	58.195415	77.1852397
OP11	57.28831	67.4963	12.9231573	13.36698	54.2076408	74.27552
OP3	66.522	76.53397	9.84735109	11.29901	62.5261898	83.0140676
OP8	65.78587	76.62295	9.83698236	10.50061	61.7826618	83.5798637
OP9	70.105	82.92051	9.13374167	11.79242	66.901124	87.7465168
pMC2A384	53.55482	62.22591	14.3521595	12.55814	49.10299	72.7574751
pSL4	67.99118	76.6455	14.8674855	10.27035	64.2571961	83.9195407
SAR406	69.24822	79.92604	5.95032987	12.05194	64.9031391	85.5864185
Synergistetes_bacterium_JGI	70.47734	86.40193	10.5094264	11.35178	68.2711592	89.6911352
Thaumarchaeota_archaeon_SCGC	70.6422	79.5107	14.9847095	7.03364	67.5840979	84.8623853
Thermotogae_bacterium_JGI	72.88344	79.6319	10.797546	10.79755	68.0981595	84.6625767
WS1	68.64344	74.20311	6.22683469	7.9318	60.9340252	84.2846553
WS3	64.59024	76.00663	7.94252329	10.43739	60.4847624	82.5359229
WWE1	66.9948	76.28686	5.89723538	9.24146	62.5097751	82.4370946

Table S2: Assignment of sequences into each category of fold, Pfam, disorder as well as transmembrane is shown for the AAA dataset.

Phyla	*% Fold*	*% Pfam*	*% Disorder*	*% TM*	*% Overlap – Fold and Pfam*	*% Assigned*
OD1	59.869591	68.0497925	7.46887967	7.61707172	55.9425015	78.2305868
OD1-i	56.3132016	66.6481121	7.50533445	8.52583728	53.1032563	76.1944522
OP11	54.8912072	63.7317437	8.72131148	8.25633383	50.8912072	74.9388972
BD1-5	46.4684534	50.3489671	3.41987716	5.82077052	40.9826912	63.5399218
WWE3	52.2198732	62.3678647	7.61099366	8.72848082	48.5049834	74.1165811
PER	53.0526834	60.807484	6.45002462	5.61299852	49.581487	71.4426391

Table S3: Assignment of sequences into each category of fold, Pfam, disorder as well as transmembrane is shown for the GEBA dataset.

Tax ID	*% Fold*	*% Pfam*	*% Disorder*	*% TM*	*% Overlap – Fold and Pfam*	*% Assigned*
446465	71.3259308	80.3396473	5.35597649	3.69039843	64.3370346	89.1900718
446466	66.1584536	73.3732644	5.79907433	2.34141029	57.6640348	85.0530901
446469	68.490566	76.6037736	6.41509434	3.0458221	60.5390836	87.3315364
469378	71.5129151	85.1660517	4.2804428	3.91143911	67.6752768	91.3653137
469382	57.4730354	64.0472522	10.8628659	2.49101181	50.4108885	79.0703647
469383	72.7841678	76.3193505	5.26048714	2.21583221	64.2760487	88.0581867
471852	65.6914894	72.3199673	7.83551555	1.7594108	56.7716858	85.7201309
471853	73.0631704	78.4743743	4.14779499	1.62097735	64.5053635	89.2252682
471854	65.0184114	71.2958092	4.89216202	1.9288094	56.2861652	83.0966158
471855	65.2727273	75.9272727	4.54545455	3.34545455	59.2727273	84.4363636
471856	65.250501	76.1923848	5.250501	1.84368737	59.5190381	85.2104208
471857	67.1891327	77.507837	7.18390805	2.32497388	60.815047	87.3301985
478801	66.4559463	76.9656262	6.51916239	3.20031608	59.6207033	86.8826551
479431	65.0204958	74.2533672	6.07066172	1.95198126	57.5639274	84.9502245
479433	66.7414974	70.8160287	4.63576159	1.75103828	58.0087552	82.7702323
479434	69.6341112	78.1042927	4.81129358	1.81503889	63.3535004	87.4099683
479437	65.1604453	78.2907662	4.28945645	1.80091683	59.9869024	85.4944335
485914	58.7251499	63.8687283	9.87693279	0.88355948	51.3726728	78.5736825
485915	69.287169	81.7107943	4.23625255	2.52545825	64.0325866	89.4501018
485917	67.8512591	74.41751	4.0480113	1.52977171	59.4492822	85.102377
485918	63.1564285	69.3689929	4.09527789	1.61582393	55.0912383	80.1086502
504472	61.7300131	69.0694626	5.08227756	1.99504878	53.3420708	80.661133
518766	69.2065711	77.7001049	4.40405453	1.88745194	61.5519049	87.4868927
519442	59.6397598	65.8772515	8.23882588	1.03402268	51.6010674	79.3195464
521095	71.8403548	81.6703622	3.91722099	2.14338507	66.518847	89.135255
521096	67.2901679	76.498801	5.27577938	2.23021583	59.5443645	86.9064748
521097	60.2487333	71.3035468	5.89590051	2.11883924	54.0764625	82.3122985
521098	64.1386074	74.3707094	5.03432494	2.2556391	57.9274273	83.1971232
522772	70.4584626	79.7311272	3.48155808	2.41296105	64.3571182	87.6249569
523794	61.4517583	73.264202	7.57439134	1.62308386	55.5455365	83.4986474
525897	69.3656825	81.6427945	3.09850921	2.45542239	64.220988	88.4828997
525898	67.9447169	79.447169	2.8087383	2.719572	62.5055729	86.4913063
525903	72.5964306	86.1830743	4.02993667	2.24525043	67.8756477	92.1704088
525904	72.3735409	79.2713123	3.92642377	1.41492748	66.2186063	87.6901309
525909	70.3875969	80.2583979	4.80620155	2.01550388	64.8062016	88.5788114
525919	68.6908078	80.9470752	7.63231198	1.7270195	63.2869081	89.9164345
526218	59.0685067	68.3834401	7.61458847	2.06998521	52.9571217	80.9265648
526224	61.0323519	70.1926572	4.58015267	1.56306798	53.8349691	79.7891676
526225	64.8175182	73.472367	7.88321168	2.50260688	57.3305527	86.5067779
526226	66.710642	75.5716799	5.91468777	1.42919965	59.2568162	86.6534741
526227	68.9207195	75.0499667	3.89740173	1.83211193	61.1925383	84.8434377

Table S4: Assignment of sequences into each category of fold, Pfam, disorder as well as transmembrane is shown for the HMP dataset.

Genomes	*% Fold*	*% Pfam*	*% Disorder*	*% TM*	*% Overlap – Fold and Pfam*	*% Assigned*
Alistipes_putredinis_DSM_17216	48.0886031	58.5923544	7.14540907	8.32440157	45.1947124	65.3090389
Anaerobaculum_hydrogeniformans_ATCC_BAA-1850	65.8841941	81.885759	24.2175274	10.6416275	63.8888889	85.5633803
Anaerococcus_hydrogenalis_DSM_7454	62.6471974	76.3542157	32.2185586	13.4715026	60.433349	83.0428639
Anaerofustis_stercorihominis_DSM_17244	64.2167157	78.1184376	32.2973541	13.4817304	62.1587568	84.5863083
Anaerostipes_caccae_DSM_14662	63.8231469	77.4772432	26.6319896	12.2496749	61.9245774	82.4707412
Anaerostipes_hadrus_comb._nov	64.2179262	79.1212654	28.0492091	13.3919156	62.741652	84.0070299
Anaerotruncus_colihominis_DSM_17241	49.5339547	62.3834887	15.9786951	9.58721704	47.1593431	67.6431425
Aneurinibacillus_aneurinilyticus_ATCC_12856	51.3655966	65.9479316	18.3836448	11.2921259	49.6566044	70.7874142
Bacteroides_caccae_ATCC_43185	61.5072317	74.5242324	28.9520426	9.89596549	58.7921847	80.8424258
Bacteroides_capillosus_ATCC_29799	49.8471571	62.6452007	18.0558386	10.7397595	47.6258406	68.2086815
Bacteroides_cellulosilyticus_DSM_14838	60.1559792	71.1785095	27.8856153	8.75216638	56.2564991	78.3362218
Bacteroides_clarus_YIT_12056	62.3123123	74.8348348	28.048048	10.3003003	59.0990991	81.021021
Bacteroides_coprocola_DSM_17136	51.2972235	64.4742831	23.6003641	8.28402367	48.3614019	71.1879836
Bacteroides_coprophilus_DSM_18228	53.8165984	65.2663934	23.6680328	8.81147541	50.2817623	72.5922131
Bacteroides_dorei_DSM_17855	57.2676728	70.8101668	25.8141382	8.49880858	54.4876886	76.7474186
Bacteroides_eggerthii_DSM_20697	59.2199522	72.1676837	27.1159459	9.60466967	56.6463253	78.4823561
Bacteroides_finegoldii_DSM_17565	55.3873162	68.1369322	27.2328286	8.58020628	51.6348475	76.1465877
Bacteroides_fluxus_YIT_12057	59.0110442	71.937751	26.6817269	9.4126506	56.3253012	77.6606426
Bacteroides_intestinalis_DSM_17393	61.5014051	74.7892413	30.0481734	9.65475713	57.8081092	81.633882
Bacteroides_ovatus_ATCC_8483	57.8685613	69.964476	29.4316163	8.22380107	54.3872114	76.69627
Bacteroides_pectinophilus_ATCC_43243	56.2405102	68.6911631	22.7755846	12.0558761	53.0822958	76.8600061
Bacteroides_plebeius_DSM_17135	58.6026852	71.3078071	26.9766285	9.32371954	55.7931377	77.4738936
Bacteroides_stercoris_ATCC_43183	55.7982319	68.6427457	27.1450858	8.91835673	52.8601144	75.3770151
Bacteroides_uniformis_ATCC_8492	53.6688518	66.0393318	23.8316769	8.88137027	51.406217	71.0721083
Bifidobacterium_adolescentis_L2-32_	52.952913	63.0087789	26.6560255	12.4900239	50.9976057	68.9146049
Bifidobacterium_angulatum_DSM_20098	63.9226519	76.8508287	31.6022099	14.8066298	62.320442	81.6574586
Bifidobacterium_bifidum_ATCC_29521	58.2909931	69.0993072	30.7159353	10.9006928	55.704388	76.0277136
Bifidobacterium_breve_DSM_20213	58.9510186	70.1343736	25.834417	14.1309059	56.8270481	76.1161682
Bifidobacterium_catenulatum_DSM_16992	59.6412556	71.2007972	28.2012955	13.4030892	57.8475336	76.5819631
Bifidobacterium_dentium_ATCC_27678	60.4883907	71.4971978	28.3426741	16.3730985	58.9271417	77.9423539
Bifidobacterium_gallicum_DSM_20093	54.0171804	65.4370894	24.6589186	11.1167256	52.5517938	70.5406771
Bifidobacterium_pseudocatenulatum_DSM_20438	60.2523659	71.2032447	27.3546643	14.511041	58.3596215	76.6561514
Blautia_hansenii_VPI_C7-24	59.1384615	72.6461538	23.9076923	12.6461538	56.9538462	78.4307692
Blautia_hydrogenotrophica_DSM_10507	61.2312389	73.3909947	22.284406	12.2360722	59.1198168	77.9445434
Blautia_sp._KLE_1732	58.0443286	72.4380704	25.0847458	11.5254237	56.2190352	77.2359844
Butyrivibrio_crossotus_DSM_2876	61.4296936	74.3912019	30.5970149	12.6080126	58.9945012	82.8750982
Cedecea_davisae_DSM_4568	66.0065333	82.8705594	14.2507146	13.9240506	64.965292	84.7692936
Cetobacterium_somerae_ATCC_BAA-474	67.8832117	82.2826808	31.0218978	15.8261447	65.4943597	87.8234904
Citrobacter_youngae_ATCC_29220	62.0606733	80.2071772	13.0595634	13.8734739	61.3577506	81.6130226
Clostridium_asparagiforme_DSM_15981	59.5376563	71.2852573	18.595522	13.3178249	56.6879907	77.1009014
Clostridium_bartlettii_DSM_16795	64.9043478	80.4869565	31.2	13.6	63.2	85.6
Clostridium_bolteae_ATCC_BAA-613	54.2550299	67.3191952	18.9233279	12.4116368	52.3518216	71.8733007
Clostridium_celatum_DSM_1785	63.7198622	78.9609644	28.7600459	13.7772675	61.5097589	84.2422503

Clostridium_difficile_70-100-2010_	67.4307036	84.4083156	30.9434968	15.3518124	66.0447761	89.3390192
Clostridium_hathewayi_DSM_13479	61.5320796	72.5940265	19.7594027	13.8136062	58.1028761	79.1897124
Clostridium_hiranonis_DSM_13275	63.8140747	80.4952215	36.0990443	14.0747176	62.0764553	86.4900087
Clostridium_hylemonae_DSM_15053	69.079465	82.8743771	26.5407815	14.3718857	67.3747705	87.4901652
Clostridium_leptum_DSM_753	49.4853126	59.10118	17.8257595	9.31458699	47.3010294	63.5701732
Clostridium_methylpentosum_DSM_5476	46.3266852	56.4756375	16.0565514	8.45746024	44.2565009	61.1966675
Clostridium_nexile_DSM_1787	53.9446125	68.9550849	22.9462416	11.100768	51.0821503	75.1919944
Clostridium_ramosum_DSM_1402	68.705371	80.937597	16.5787023	15.0263893	66.6563179	85.4393046
Clostridium_scindens_ATCC_35704	57.7430386	71.8124084	21.9101124	11.333659	55.4958476	76.4044944
Clostridium_sp_M62-1_	51.4972884	66.18722	25.8193822	10.7993398	49.6345202	71.799104
Clostridium_sp._SS2-1_	61.8271301	76.8071363	27.6837896	12.6730237	60.2276223	82.0055368
Clostridium_spiroforme_DSM_1552	63.9479905	78.7628054	21.6312057	12.6477541	62.4113475	82.8605201
Clostridium_sporogenes_ATCC_15579	64.2026363	80.9253037	32.4373223	15.3528043	62.4450762	85.9912122
Collinsella_aerofaciens_ATCC_25986	61.3114754	72.3360656	25.6557377	10.5737705	58.8934426	79.2622951
Collinsella_intestinalis_DSM_13280	61.5384615	71.8851571	24.0520043	13.5427952	59.5341278	77.0855905
Collinsella_stercoris_DSM_13279	55.5512573	66.4990329	22.0116054	11.450677	53.384913	72.2630561
Coprococcus_comes_ATCC_27758	53.5480624	67.7906392	20.1811777	11.1977856	51.0065425	73.4272773
Coprococcus_eutactus_ATCC_27759	57.5530179	70.0163132	28.319739	12.1044046	55.1712887	77.1941272
Corynebacterium_ammoniagenes_DSM_20306	64.5710059	79.5118343	24.704142	15.8284024	63.0547337	84.0976331
Desulfitobacterium_hafniense_DP7_	61.9684751	75.8247801	16.6605572	13.3797654	60.0623167	79.8753666
Desulfovibrio_piger_ATCC_29098	49.1514771	61.5336266	17.0961659	10.5593966	47.3601508	66.813325
Dorea_formicigenerans_ATCC_27755	59.1587112	74.1945107	25.5369928	12.4105012	57.0107399	79.5644391
Dorea_longicatena_DSM_13814	61.7405583	76.8472906	27.7504105	13.3661741	59.7701149	81.6748768
Edwardsiella_tarda_ATCC_23685	59.8969831	77.3117488	9.81113564	12.803532	58.7687025	79.2985038
Enterobacter_cancerogenus_ATCC_35316	65.6974294	83.4176148	15.2549515	13.990729	65.002107	84.8082596
Enterococcus_faecalis_TX1467_	59.8538094	75.1194827	16.6713523	13.4101771	57.6047231	80.9671071
Enterococcus_faecalis_TX2134_	60.5101414	76.5519361	20.6822372	14.4437615	58.9121082	81.8070068
Enterococcus_faecalis_TX2137_	62.001308	79.3328973	20.3400916	15.206017	60.9548725	83.4532374
Enterococcus_faecalis_TX4244_	63.7779282	79.9593771	20.4468517	15.4705484	62.4238321	84.563304
Escherichia_coli_MS_107-1	60.5327791	80.3136218	13.9429435	12.8471566	59.6825997	82.0139807
Escherichia_coli_MS_110-3	60.0903342	80.0542005	14.2547425	12.7190605	58.9521229	82.1499548
Escherichia_coli_MS_115-1	62.1200312	81.7225253	14.8480125	12.9579111	61.1652377	83.4957132
Escherichia_coli_MS_116-1	60.5228263	79.8446676	13.9230915	12.9191135	59.3104755	81.9473385
Escherichia_coli_MS_117-3	59.2349727	79.5628415	14.5355191	12.4954463	58.2695811	81.6757741
Escherichia_coli_MS_119-7	59.7151205	80.0036523	13.8970051	12.2899927	58.5098612	82.0672023
Escherichia_coli_MS_124-1	57.2386717	77.637821	14.0029445	11.5655161	56.0117782	79.8135122
Escherichia_coli_MS_145-7	58.9611771	79.4539975	14.5896118	12.143237	58.0216274	81.3508243
Escherichia_coli_MS_146-1	62.0512821	81.7751479	14.1814596	13.1952663	61.183432	83.4122288
Escherichia_coli_MS_153-1	59.9964138	79.5768334	14.3266989	12.5694818	58.7771203	81.6926663
Escherichia_coli_MS_16-3	60.5599852	80.1038383	14.0181717	13.0725014	59.5216021	82.0508066
Escherichia_coli_MS_175-1	62.5025065	81.8327652	14.1768598	13.2745137	61.4597955	83.4970924
Escherichia_coli_MS_182-1	60.062546	79.874908	14.0360559	12.6931567	59.1979397	81.7328918
Escherichia_coli_MS_185-1	61.5820276	81.0269964	14.2722296	13.3094204	60.4870682	83.0281291
Escherichia_coli_MS_187-1	64.7480452	83.6012163	13.7054735	13.9661164	63.835795	85.0781929
Escherichia_coli_MS_196-1	58.1407563	77.6085434	13.4278711	12.289916	57.1078431	79.7268908
Escherichia_coli_MS_198-1	59.3853676	80.4784355	14.1086083	12.3450323	58.4075432	82.5388511
Escherichia_coli_MS_200-1	60.4809104	79.6622614	14.2070485	12.8487518	59.6365639	81.6629956

Escherichia_coli_MS_21-1	58.713091	78.4092849	14.5417307	12.1693122	57.4671446	80.9011777
Escherichia_coli_MS_45-1	61.4464586	80.7699495	13.866567	13.081667	60.4746776	82.6387591
Escherichia_coli_MS_57-2	60.5662854	80.911307	14.5134071	13.0320645	59.5724733	82.8989312
Escherichia_coli_MS_60-1	58.2493922	78.1174019	14.2063216	12.2264675	57.3115665	80.3056617
Escherichia_coli_MS_69-1	60.178731	80.1608579	14.6738159	12.6541555	59.2493298	82.0911528
Escherichia_coli_MS_78-1	62.282247	81.6402427	14.190644	13.4468585	61.3231552	83.4801331
Escherichia_coli_MS_79-10	60.0630096	79.6515938	13.4358784	12.6204596	59.0993328	81.5048184
Escherichia_coli_MS_84-1	58.4462356	78.9058481	14.4572115	12.1762991	57.451552	80.8780655
Escherichia_coli_MS_85-1	56.7664281	77.9603123	14.1997398	11.5159401	55.6929083	80.0422902
Eubacterium_biforme_DSM_3989	59.9534342	76.0962359	19.9456733	13.5816841	58.3624369	81.2960807
Eubacterium_cylindroides_ATCC_27803	64.8895293	78.7704131	19.4044188	13.3045149	63.0163305	84.2459174
Eubacterium_dolichum_DSM_3991	58.5070892	72.852377	16.0967473	13.0525438	56.6305254	77.9816514
Eubacterium_hallii_DSM_3353	59.7209584	73.3697301	26.2966333	10.4337276	57.840461	78.7382469
Eubacterium_siraeum_DSM_15702	55.0902527	67.9783394	26.6425993	12.1299639	53.3574007	75.198556
Eubacterium_ventriosum_ATCC_27560	57.538036	69.8478562	31.0511757	12.3443983	54.8063624	77.1438451
Faecalibacterium_cf._prausnitzii_KLE1255	50.6444054	65.2606913	15.5536028	10.7791447	48.7990627	70.0351494
Flavonifractor_plautii_ATCC_29863	53.4256055	67.0588235	16.8627451	11.2341407	51.2572088	72.1107266
Hafnia_alvei_ATCC_51873	60.733614	79.6151533	13.7101624	13.3092804	59.6512327	81.8801363
Helicobacter_pylori_GAM100Ai_	56.2573099	73.2748538	18.1871345	10	54.502924	78.128655
Helicobacter_pylori_GAM101Biv_	56.93302	73.3254994	17.920094	9.98824912	55.052879	77.9083431
Helicobacter_pylori_GAM103Bi_	55.9411765	72.8235294	17.9411765	10.1176471	54.1176471	77.8235294
Helicobacter_pylori_GAM105Ai_	55.2721088	71.8253968	18.537415	9.75056689	53.1746032	77.2108844
Helicobacter_pylori_GAM112Ai_	55.7395774	72.0159909	18.3894917	9.82295831	53.7407196	76.92747
Helicobacter_pylori_GAM114Ai_	56.5704184	73.1879788	18.0907484	10.1944608	54.9204478	77.9611078
Helicobacter_pylori_GAM115Ai_	54.5709478	71.0600112	17.9472799	9.59057768	52.9444756	75.9394279
Helicobacter_pylori_GAM117Ai_	56.0623557	72.4018476	18.0715935	9.98845266	53.8683603	77.5404157
Helicobacter_pylori_GAM118Bi_	56.0961649	72.9822553	18.9467659	10.1316543	54.4361763	77.847739
Helicobacter_pylori_GAM119Bi_	55.1704545	71.875	18.6363636	9.82954545	53.2954545	77.3295455
Helicobacter_pylori_GAM120Ai_	55.4354013	72.3392146	19.1804212	9.73249858	53.8417758	77.1770063
Helicobacter_pylori_GAM121Aii_	55.6002298	72.5445146	18.322803	9.76450316	53.8770821	77.3118897
Helicobacter_pylori_GAM201Ai_	57.0668244	73.9207569	17.5635719	9.99408634	55.4109994	78.1194559
Helicobacter_pylori_GAM210Bi_	56.2828755	72.4722385	17.7089421	10.1694915	54.6464056	77.3816482
Helicobacter_pylori_GAM231Ai_	56.4449676	73.8669806	17.4220129	10.1236021	55.3266627	77.8104768
Helicobacter_pylori_GAM239Bi_	56.4431487	73.4110787	18.0758017	9.91253644	54.8104956	77.8425656
Helicobacter_pylori_GAM244Ai_	56.649092	73.3450498	17.2231986	10.0175747	55.0087873	77.6215583
Helicobacter_pylori_GAM245Ai_	56.152513	72.3859041	18.7752744	9.99422299	54.1883304	77.3541306
Helicobacter_pylori_GAM246Ai_	56.2678878	73.1539782	18.7178019	9.84544934	54.4934173	78.0194619
Helicobacter_pylori_GAM249T_	56.8489124	73.5449735	17.8130511	10.2880658	55.085244	78.4832451
Helicobacter_pylori_GAM250AFi_	57.4442435	74.0807715	17.2393008	10.3074141	55.575648	78.6015672
Helicobacter_pylori_GAM250T_	57.2115385	73.9783654	17.1274038	10.2764423	55.3485577	78.4855769
Helicobacter_pylori_GAM252Bi_	57.4879227	74.2149758	17.3309179	10.326087	55.615942	78.8647343
Helicobacter_pylori_GAM252T_	57.358263	74.5476478	17.1290712	10.3739445	55.4885404	79.0711701
Helicobacter_pylori_GAM254Ai_	56.3983787	72.6693688	17.8922988	10.075275	54.2559351	77.6491025
Helicobacter_pylori_GAM260ASi_	57.519017	73.6102984	17.612639	10.1228789	55.2954944	78.7009947
Helicobacter_pylori_GAM260Bi_	55.9701493	73.0769231	18.0826636	9.93111366	54.4202067	77.8989667
Helicobacter_pylori_GAM260BSi_	57.3141487	74.1007194	18.2254197	9.89208633	55.6954436	78.177458
Helicobacter_pylori_GAM263BFi_	56.4281559	73.0075625	18.4991274	10.122164	54.9156486	77.7196044

Helicobacter_pylori_GAM264Ai_	57.2700297	73.3531157	17.6261128	10.0296736	55.6676558	77.7448071
Helicobacter_pylori_GAM265BSii_	54.9544419	71.6400911	17.881549	9.90888383	53.1321185	76.4236902
Helicobacter_pylori_GAM268Bii_	56.4867968	72.3880597	17.2789897	9.98851894	54.3628014	77.4971297
Helicobacter_pylori_GAM270ASi_	56.622325	73.3371891	18.2186235	10.1214575	54.7715442	78.1376518
Helicobacter_pylori_GAM42Ai_	56.5090485	72.6211325	18.8558085	10.0992411	54.4074723	77.7583187
Helicobacter_pylori_GAM71Ai_	56.4629847	72.7967098	17.4500588	10.0470035	54.8766157	77.3795535
Helicobacter_pylori_GAM80Ai_	56.8834212	73.4036321	18.0433509	9.90041008	55.0087873	77.9144698
Helicobacter_pylori_GAM83Bi_	56.8155112	72.8554642	17.9788484	9.98824912	54.7003525	77.6733255
Helicobacter_pylori_GAM83T_	57.0754717	73.0542453	17.865566	10.0235849	54.7759434	78.0070755
Helicobacter_pylori_GAM93Bi_	56.491633	72.9371033	17.5995384	9.92498557	54.4720138	78.2458165
Helicobacter_pylori_GAM96Ai_	55.0997151	72.0797721	17.1509972	9.51566952	53.4472934	76.6381766
Helicobacter_pylori_GAMchJs106B_	57.3770492	74.195507	17.911354	10.1396478	55.7984214	78.3242259
Helicobacter_pylori_GAMchJs114i_	56.6037736	73.4669811	17.7476415	10.0235849	54.8938679	77.6533019
Helicobacter_pylori_GAMchJs117Ai_	57.5104728	74.1472172	18.491921	10.2333932	55.8348294	78.39617
Helicobacter_pylori_GAMchJs136i_	55.7659208	72.5760184	18.4165232	9.9827883	54.1594951	77.6247849
Helicobacter_pylori_HP116Bi_	56.1596298	72.9323308	18.507808	9.89010989	54.3666859	77.6171197
Helicobacter_pylori_HP250AFii_	57.2932331	74.1353383	17.0827068	10.2857143	55.4285714	78.6766917
Helicobacter_pylori_HP250AFiii_	57.3405535	74.0673887	17.0276775	10.2888087	55.4753309	78.5800241
Helicobacter_pylori_HP250AFiV_	57.4442435	73.960217	17.1187462	10.3074141	55.4550934	78.6015672
Helicobacter_pylori_HP250ASi_	57.09997	73.9117382	16.9918943	10.3272291	55.2386671	78.4148904
Helicobacter_pylori_HP250ASii_	57.0828331	73.8895558	17.0468187	10.3241297	55.2220888	78.3913565
Helicobacter_pylori_HP250BFi_:	56.4206856	72.9808251	16.3858222	10.0522952	54.4450901	77.5130738
Helicobacter_pylori_HP250BFii_:	57.2716346	74.0985577	17.1875	10.3365385	55.46875	78.5456731
Helicobacter_pylori_HP250BFiii_	57.1085783	73.905219	17.2165567	10.2579484	55.3089382	78.4043191
Helicobacter_pylori_HP250BFiV_	56.8195354	73.8534842	16.9148303	10.244193	54.9136391	78.3204288
Helicobacter_pylori_HP250BSi_	57.2285543	73.965207	17.0965807	10.3179364	55.3689262	78.5842831
Helicobacter_pylori_HP260AFi_:	57.2594752	73.1195335	17.4927114	10.0291545	55.1020408	78.1341108
Helicobacter_pylori_HP260AFii_:	56.993007	73.1351981	17.5407925	10.02331	54.95338	78.030303
Helicobacter_pylori_HP260ASii_	56.9355775	72.9541497	17.5856065	9.98258851	54.8461985	77.945444
Helicobacter_pylori_HP260BFii_	57.3819486	73.6999402	18.1709504	9.92229528	55.5289898	78.0633592
Helicobacter_pylori_HP260Bi_	56.091954	73.045977	18.3908046	9.94252874	54.5977011	77.816092
Holdemania_filiformis_DSM_12042	55.9456929	69.1713483	13.5299625	13.1554307	53.7453184	74.5552434
Klebsiella_sp._MS_92-3	63.4432824	78.9058729	11.5848753	13.4513274	62.1721641	81.2711183
Lactobacillus_rhamnosus_ATCC_21052	60.548661	73.2201176	15.1534944	16.0352711	58.9810581	78.1841933
Listeria_innocua_ATCC_33091	68.0751174	84.3393696	23.5412475	15.1576123	67.0020121	87.7263581
Marvinbryantia_formatexigens_I-52_	55.4233871	67.8427419	22.1975806	12.5806452	53.2056452	73.2459677
Methanobrevibacter_smithii_DSM_2374	63.0646083	79.1881075	27.3299028	10.920526	61.5208691	84.1623785
Methanobrevibacter_smithii_DSM_2375	63.6727689	78.4897025	27.3455378	10.812357	61.3844394	83.9244851
Mitsuokella_multacida_DSM_20544	63.2352941	75.678733	22.1719457	11.9532428	61.387632	79.147813
Parabacteroides_johnsonii_DSM_18315	58.6191829	71.1601486	25.409657	9.0670745	55.4293205	77.4961765
Parabacteroides_merdae_ATCC_43184	54.216059	65.3992395	24.7148289	8.25318721	51.5544621	70.5435026
Paraprevotella_clara_YIT_11840	52.0746379	62.5583108	26.9825681	8.1757918	48.1708814	70.1203044
Paraprevotella_xylaniphila_YIT_11841	55.7142857	66.2571429	27.9428571	8.88571429	52.1714286	73.2285714
Parasutterella_excrementihominis_YIT_11859	62.3487544	76.3345196	26.8683274	12.0640569	60.6761566	80.8185053
Peptostreptococcus_micros_ATCC_33270	60.8241439	76.9587928	31.9210679	15.6703424	58.9088799	82.9367382
Prevotella_copri_CB7_	50.2929115	63.884007	23.4622144	7.79144698	47.71529	69.9179848
Prevotella_stercorea_DSM_18206	50.4240052	62.9810828	26.4187867	8.44748858	47.3581213	70.6131768

Proteus_penneri_ATCC_35198	60.4254465	74.7742324	9.29159141	10.0140478	56.1107766	80.272928
Providencia_alcalifaciens_DSM_30120	61.5516818	80.3093543	15.0012276	12.6442426	60.1767739	83.4765529
Providencia_rettgeri_DSM_1131	61.9410674	78.9614671	13.9501339	12.9198434	60.8695652	81.805069
Providencia_rustigianii_DSM_4541	62.9044586	82.522293	15.2101911	13.2993631	61.6815287	85.0955414
Providencia_stuartii_ATCC_25827	59.9823711	78.8893786	14.081093	12.494491	58.704275	81.9964742
Roseburia_intestinalis_L1-82_	55.2345059	67.7763819	22.8433836	11.2646566	51.9891122	74.7487437
Roseburia_inulinivorans_DSM_16841	54.512958	69.0795353	20.8221626	11.8409294	51.6979446	75.6255585
Ruminococcus_gnavus_ATCC_29149	55.0979407	68.2320442	22.5263687	11.752888	52.963335	73.8071321
Ruminococcus_lactaris_ATCC_29176	57.9097049	72.2360469	27.301813	10.9847138	56.1677924	77.1773907
Ruminococcus_obeum_ATCC_29174	53.8679245	67.1698113	22.1226415	10.3537736	51.4622642	72.9009434
Ruminococcus_torques_ATCC_27756	55.7763557	68.2721455	27.3155945	10.1044123	53.6207477	73.358033
Subdoligranulum_variabile_DSM_15176	58.5797101	71.2463768	16.173913	13.6231884	55.942029	77.2753623
Succinatimonas_hippei_YIT_12066	63.3378318	79.4871795	20.062978	13.7201979	62.2132254	82.6810616
Sutterella_parvirubra_YIT_11816	58.745098	70.0784314	28.3137255	11.6078431	56.5490196	77.2941176
Yokenella_regensburgei_ATCC_43003	66.2148071	84.9217935	13.3680918	14.5985401	65.3180396	86.5693431

Table S5: Assignment of sequences into each category of fold, Pfam, disorder as well as transmembrane is shown for the *E. coli* pangenome.

Genomes	***% Fold***	***% Pfam***	***% Disorder***	***% TM***	***% Overlap – Fold and Pfam***	***% Assigned***
NC_000913	72.6570048	94.7826087	3.38164251	15.6038647	72.0531401	95.942029
NC_002655	65.5694287	89.0087022	4.99432463	12.9776769	64.226258	91.3166856
NC_002695	65.56495	89.5465027	5.07302075	12.7209839	64.3543428	91.6410453
NC_004431	62.6211782	82.3639075	4.10141685	12.9753915	61.7076808	84.1722595
NC_007779	72.6560646	94.9204842	3.48920009	15.3334916	71.8727747	96.2734394
NC_007946	64.5405621	85.8879809	4.066175	13.6934423	63.583815	87.7815428
NC_008253	69.7986577	90.4524789	3.72374973	14.6135527	68.88937	91.9896081
NC_008563	70.4063205	91.8961625	4.55981941	15.3498871	69.6613995	93.7923251
NC_009800	67.9012346	90.1234568	3.2007316	14.4947417	67.1925011	91.2894376
NC_009801	66.0981259	86.9446199	3.97978522	13.7292061	65.2137292	88.3975574
NC_010468	72.8983091	95.3560372	3.88187664	15.6227673	72.2314837	96.5944272
NC_010473	72.599418	94.8351115	3.56450048	15.4219205	71.8477207	96.1930165
NC_010498	68.4521299	89.1396035	3.75369043	14.5719106	67.6507803	90.6157739
NC_011353	62.7469426	85.7196613	4.60959548	12.229539	61.6745061	87.4694262
NC_011415	67.5155147	90.4130109	4.3227049	14.3804836	66.5525358	92.0607747
NC_011601	68.2058047	91.6007036	4.13368514	14.182058	67.414248	93.2497801
NC_011741	70.2416571	91.6685846	3.70540852	15.0287687	69.5051784	92.9804373
NC_011742	68.4513652	91.5742321	4.1168942	14.4837884	67.5981229	93.3020478
NC_011745	65.2412951	89.5744248	4.78517614	13.5206679	64.426797	91.386683
NC_011748	67.4658254	91.1041009	4.54258675	14.1745531	66.6666667	92.7024185
NC_011750	70.8359788	92.4867725	4.88888889	14.1375661	70.010582	94.0740741
NC_011751	68.7279519	91.0354845	4.15023864	14.2768209	67.773397	92.7578336
NC_011993	71.2065814	93.6700183	3.70201097	15.4478976	70.2010969	95.3382084
NC_012759	72.8364795	95.3910272	3.53027703	15.5675411	72.2726158	96.4452072
NC_012892	71.898855	94.5849237	3.4351145	15.3148855	71.1354962	95.7776718
NC_012947	70.6007569	93.0227058	3.33491012	15.2081362	69.9148534	94.1343425
NC_012967	71.452381	94.3809524	3.5	15.3333333	70.7857143	95.5

NC_012971	71.7312786	94.7507826	3.53960992	15.4105466	71.0329882	95.8584156
NC_013008	64.95336	88.8254331	4.94955264	12.7355797	63.8682658	90.8242909
NC_013353	66.3168317	91.2277228	5.18811881	12.950495	65.3267327	93.0891089
NC_013361	64.0858209	90.6156716	5.05597015	12.4813433	63.0597015	92.4626866
NC_013364	66.5458937	91.5056361	5.25362319	13.1441224	65.6199678	93.2568438
NC_013654	72.4861624	93.9806273	3.48247232	15.7287823	71.7250923	95.2490775
NC_013941	66.6866267	90.4590818	4.69061876	13.4331337	65.8283433	92.2155689
NC_016902	68.7403486	92.4112067	4.32384734	14.7804986	68.1005956	93.8451357
NC_017625	72.5961538	94.0865385	3.58173077	15.5769231	71.8269231	95.4567308
NC_017626	67.5	90.7291667	4.5625	14.1041667	66.7083333	92.3958333
NC_017628	66.8630339	89.3751315	4.35514412	14.1594782	65.9793814	91.0372396
NC_017631	69.0525876	90.3797997	4.1736227	14.4824708	68.1761269	91.9449082
NC_017632	68.8387097	91.2258065	3.78494624	14.6451613	67.9569892	92.8172043
NC_017633	68.5623003	91.6506922	4.32374867	13.9936102	67.5825346	93.6102236
NC_017634	70.4673741	92.345902	3.54481824	15.2178821	69.5190788	93.9263942
NC_017635	68.7527939	92.6687528	4.31381314	14.7742512	68.0375503	94.0992401
NC_017638	71.8243719	93.8013618	3.52195351	15.1913595	70.9321437	95.2571026
NC_017641	62.6929842	84.7762361	4.02579637	12.9763533	61.7158491	86.5350791
NC_017644	65.3396265	87.2973528	4.0016417	13.9749641	64.436692	88.9595732
NC_017646	67.8378918	89.498902	4.29227391	13.7153124	66.8995808	91.1958475
NC_017651	65.4127694	85.5225702	3.72102481	13.8877593	64.6807645	86.9865799
NC_017652	65.4127694	85.5225702	3.72102481	13.8877593	64.6807645	86.9865799
NC_017656	67.3452769	90.6148208	4.43811075	13.7418567	66.3680782	92.4674267
NC_017660	69.1455359	92.0519923	3.89942468	13.9995738	68.4849776	93.3944172
NC_017663	70.1758392	91.2080384	3.83649235	14.615209	69.1482074	92.8979219
NC_017664	67.9843614	92.0286707	4.27888792	14.5742832	67.3110339	93.4404865
NC_017906	66.8718967	90.2482622	4.74677259	13.1876862	65.7199603	92.2740814
NC_018650	66.2105051	90.4206078	4.32682632	13.6647213	65.4658885	91.9702153
NC_018658	66.0233213	90.0080418	4.44310414	13.6911942	65.2191395	91.6767189
NC_018661	65.647946	90.0972415	4.46517166	13.4153602	64.8541377	91.7642389
NC_020163	69.2022668	91.4995641	3.70531822	14.646905	68.2868352	93.3086312
NC_020518	75.7832345	96.6412645	3.07648885	16.4549817	75.2751905	97.5162292
NC_022364	69.2135335	92.1129285	3.92112929	14.6986332	68.5413399	93.4349093
NC_022370	69.7891763	92.3060204	4.15127146	14.9098022	68.7676592	94.0882417
NC_022648	67.9635762	91.0596026	4.40811258	14.3004967	67.0736755	92.7152318

Table S6: Non-parametric Mann-Whitney U test on the percentage of assigned sequences between the different datasets. H_0 = There is no difference between the percentage of assigned sequences between the different (meta)genomic categories. Signifiance values at p-value 0.005 are in bold.

	MDM	HMP	AAA	GEBA	*E coli*
MDM	-	0.1083	0.0400	**4.085e^{-5}**	**2.996e^{-13}**
HMP	-	-	0.0068	**2.357e^{-16}**	**2.2e^{-16}**
AAA	-	-	-	**1.63e^{-7}**	**6.049e^{-5}**
GEBA	-	-	-	-	**1.417e^{-14}**
E. coli	-	-	-	-	-

Table S7 : Coding density for the AAA dataset

Phyla	Coding density
BD1-5	0.86002541
OD1-i	0.82929353
OD1	0.86705656
OP11	0.86205013
PER	0.86139054
WWE3	0.85900727

For coding density of "Microbial Dark Matter" see Supplementary Table 2 in Rinke *et al* 2013.

Figure S2: Histogram of sequence length (range 50 to 500) for all proteomic sequences and unassigned sequences for the HMP dataset

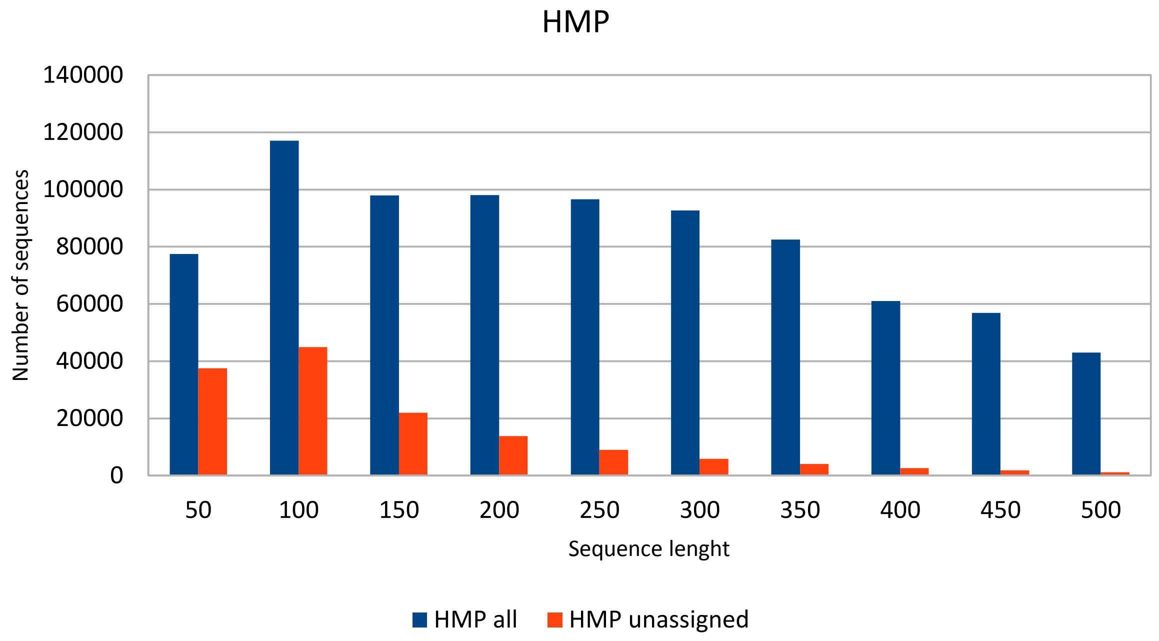

Figure S3: Histogram of sequence length (range 50 to 500) for all proteomic sequences and unassigned sequences for the AAA dataset

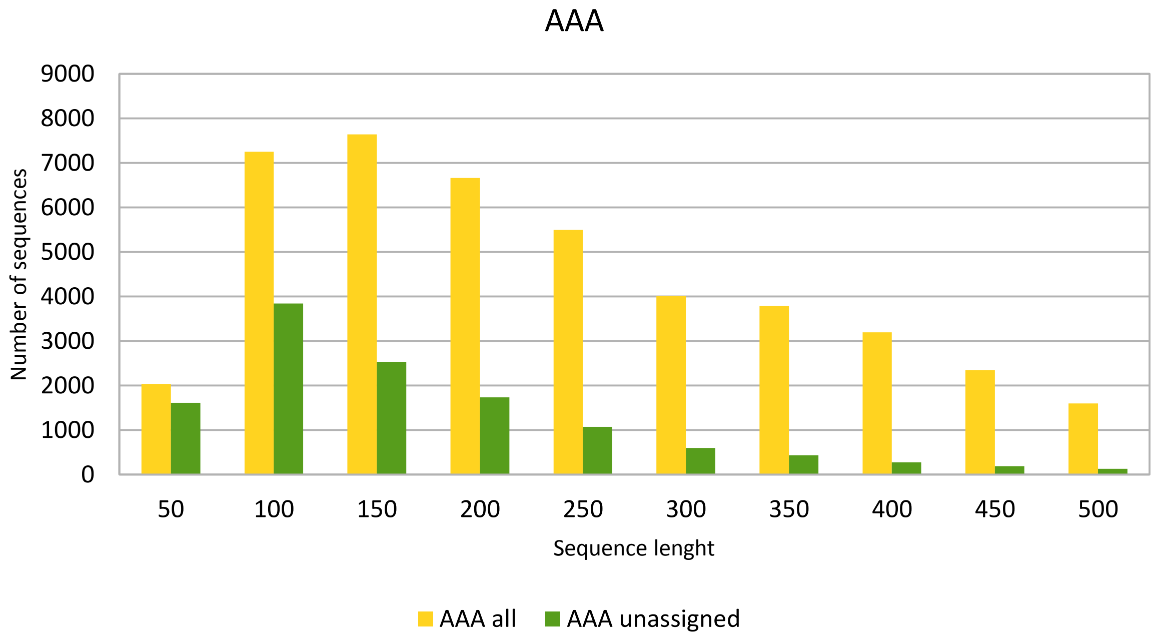

Figure S4: Histogram of sequence length (range 50 to 500) for all proteomic sequences and unassigned sequences for the GEBA dataset

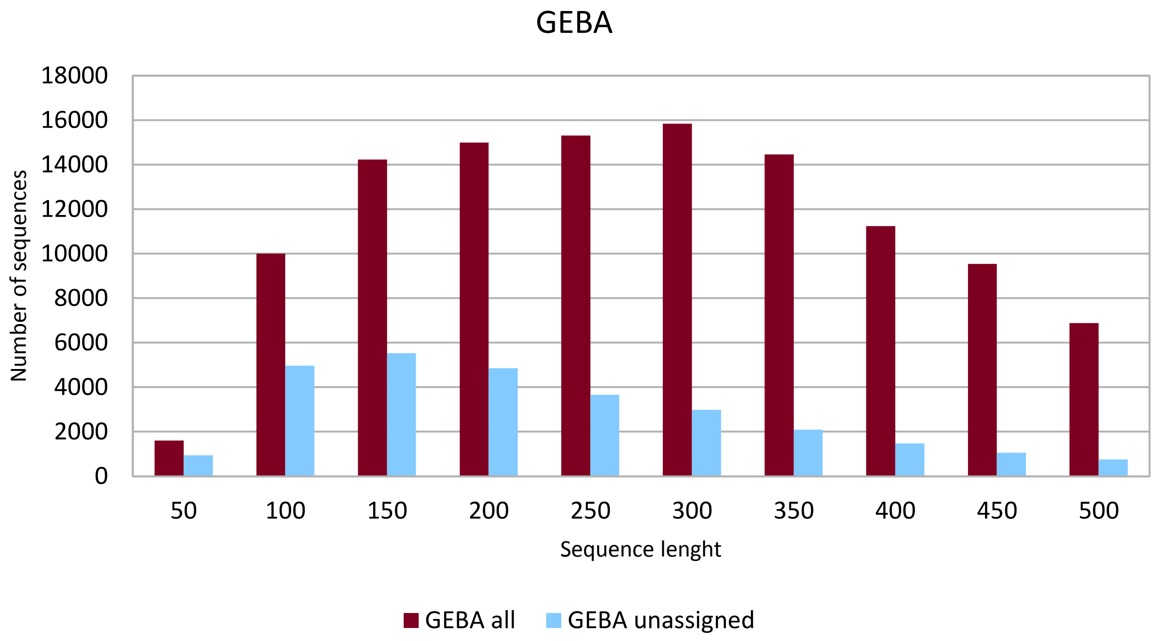

Figure S5: Histogram of sequence length (range 50 to 500) for all proteomic sequences and unassigned sequences for the MDM dataset

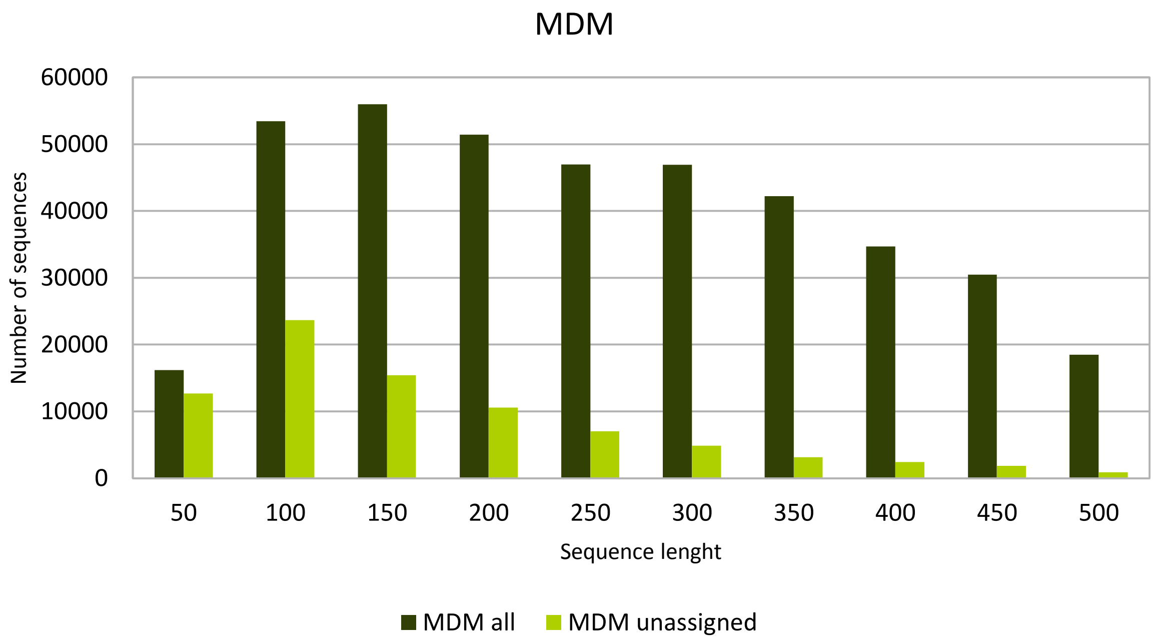

Figure S6: Histogram of sequence length (range 50 to 500) for all proteomic sequences and unassigned sequences for the E coil dataset

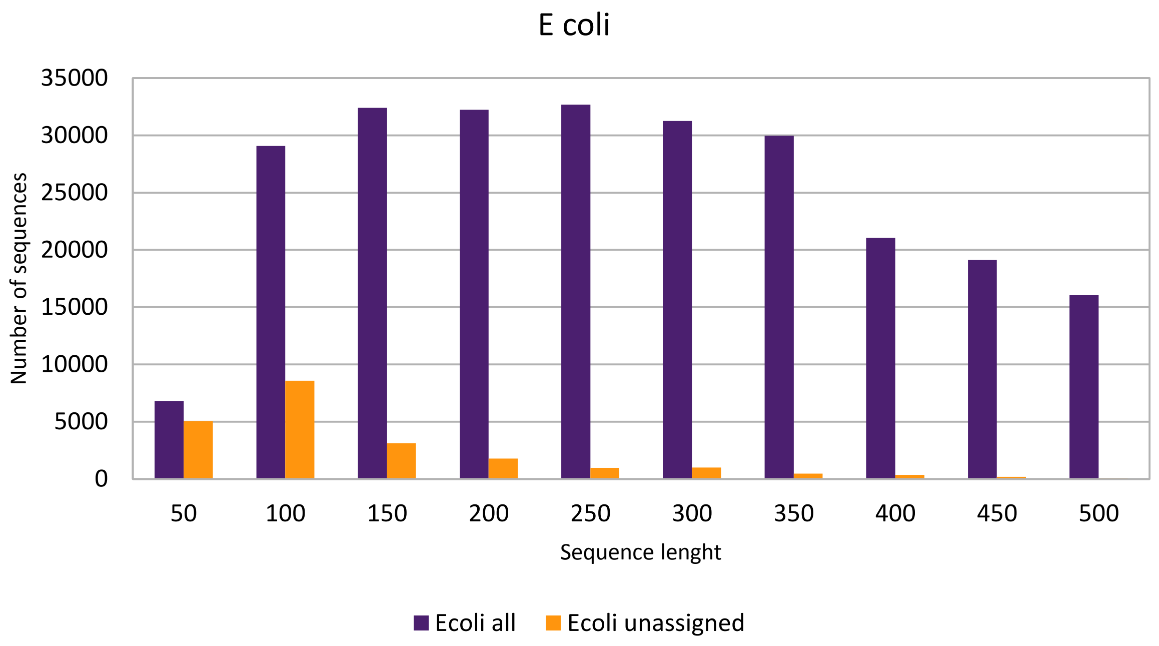

Supplement: Supplementary Information [file srep14717-s1.pdf]
